# Direction Dependent Electrical Conductivity of Polymer/Carbon Filler Composites

**DOI:** 10.3390/polym11040591

**Published:** 2019-04-01

**Authors:** Karina Kunz, Beate Krause, Bernd Kretzschmar, Levente Juhasz, Oliver Kobsch, Wolfgang Jenschke, Mathias Ullrich, Petra Pötschke

**Affiliations:** Leibniz-Institut für Polymerforschung Dresden e.V. (IPF), Hohe Str. 6, 01069 Dresden, Germany; kunz@ipfdd.de (K.K.); krause-beate@ipfdd.de (B.K.); kobsch@ipfdd.de (O.K.); wjke@ipfdd.de (W.J.); ullrich@ipfdd.de (M.U.)

**Keywords:** electrical volume conductivity, carbon fillers, CNT alignment

## Abstract

The method of measuring electrical volume resistivity in different directions was applied to characterize the filler orientation in melt mixed polymer composites containing different carbon fillers. For this purpose, various kinds of fillers with different geometries and aspect ratios were selected, namely carbon black (CB), graphite (G) and expanded graphite (EG), branched multiwalled carbon nanotubes (b-MWCNTs), non-branched multiwalled carbon nanotubes (MWCNTs), and single-walled carbon nanotubes (SWCNTs). As it is well known that the shaping process also plays an important role in the achieved electrical properties, this study compares results for compression molded plates with random filler orientations in the plane as well as extruded films, which have, moreover, conductivity differences between extrusion direction and perpendicular to the plane. Additionally, the polymer matrix type (poly (vinylidene fluoride) (PVDF), acrylonitrile butadiene styrene (ABS), polyamide 6 (PA6)) and filler concentration were varied. For the electrical measurements, a device able to measure the electrical conductivity in two directions was developed and constructed. The filler orientation was analyzed using the ratio σ_in/th_ calculated as in-plane conductivity σ_in-plane_ (σ_in_) divided by through-plane conductivity σ_through-plane_ (σ_th_). The ratio σ_in/th_ is expected to increase with more pronounced filler orientation in the processing direction. In the extruded films, alignment within the plane was assigned by dividing the in-plane conductivity in the extrusion direction (x) by the in-plane conductivity perpendicular to the extrusion direction (y). The conductivity ratios depend on filler type and concentration and are higher the higher the filler aspect ratio and the closer the filler content is to the percolation concentration.

## 1. Introduction

Electrically conductive composite materials currently play a systematically growing role for new technologies and products. In order to obtain conductive polymer composites (CPCs), various carbon fillers, such as carbon nanotubes (CNTs), carbon black (CB), and graphite (G), are used by exploiting the phenomenon of filler percolation [1,2,3,4,5,6,7,8]. Depending on the application, there are specific requirements concerning conductivity values [1], but the direction of conductance may also be of interest, meaning that different values may be required for the in-plane or through-plane of the sample or component [9]. One of the main applications of CPCs is for electrostatic discharge materials, which are used, for example, in the housings of electrical and electronic parts [10], antistatic packaging, and fuel lines, and also to reduce the explosion risk in fuel tanks and hazardous materials containers the electrical discharge ability, as needed. In these applications, the in-plane conductivity mainly at the surface of the plastic parts is required and electrical resistivity values can be in the range of moderate conductive materials [10,11,12,13]. For the replacement of metallic parts by CPC materials, e.g., for weight reduction and fuel savings in automotive applications, electrostatic painting processes, which also require the conductivity in-plane on the surface of the parts to be painted, are used [11,12]. In cable production, CPC formulations are used in some of the layers to eliminate any electrical field stresses by homogenizing the electric field around the conductor. In such cases, both in- and through-plane conductivity are required. On the other hand, CPC based bipolar plates used in fuel cells have to possess high through-plane conductivity, which is related to the electron flow direction in an operating fuel cell [14,15,16]. Moreover, when using CPCs as adhesives to join different conductive parts, through-plane conductivity is a requirement to achieve conductive joined parts.

The requirements according to the direction of the electrical conductivity can be met by adapting the composite processing and selecting the appropriate type and concentration of fillers. The most important filler parameters that influence the electrical properties of the composite materials are aspect ratio, morphology, and conductivity. The long and very thin CNTs (length~ 1–100 μm, diameter ~1–15 nm) build a percolating and electrical conducting network in a polymer matrix at much lower concentrations than more compact spherical carbon black or plate-like graphite. On the other hand, the electrical conductivity of composites filled with CB is expected to be isotropic because the aspect ratio of CB aggregates is close to 1. In contrast, the electrical conductivity of the CNT composite correlates strongly with the orientation of the CNTs in the network. During melt processing, CNTs orient in the flow direction [9,17,18]. Therefore, it is recommended to measure the electrical conductivity in extruded films and injection molded parts in 3 directions: x-in-plane in the extrusion/flow, y-in-plane perpendicular to the extrusion/flow, and the z-through-plane/thickness direction. In compression molded plates, the orientation in-plane is expected to be isotropic (x=y), so that two measurement directions, namely in-plane and through-plane, are sufficient. Higher in-plane than through-plane conductivity in the mentioned systems indicates a preferential alignment of the CNTs in-plane and can be of an advantage or disadvantage, depending on the application. In cases where only high through-plane conductivity is aimed, there are some methods for the vertical alignment of the CNTs, like using an electric or magnetic field, infiltration of the CNT forests, or in situ polymerization [9,19,20,21]. If high conductivity in both directions at a relatively low filler concentration is needed, hybrid fillers consisting of CNTs and CB particles, which usually are taken into account according to their synergistic effect on the lowering of the electrical percolation threshold p_c_, can be used [4,22,23,24,25,26]. 

At each investigation, development, or optimization step, it is needed to perform control measurements of the electrical conductivity. In the literature, no studies comparing different processing types, fillers with different morphology, and polymer matrix types according to direction dependent electrical conductivity were found. Reports comparing different fillers or polymers with respect to the electrical properties of their composites are mostly based on measurements in only one direction, or show combined percolation curves with high resistivity values measured with through-plane equipment combined with low resistivity values measured with in-plane equipment. Papers comparing electrical conductivity of composites in two or three different directions are very rare and focus mostly on one filler system. Cesano et al. [27] studied the electrical conductivity of samples based on PP with 1–7 wt% multiwalled CNT (MWCNT) manufactured by injection molding and found a strong direction dependency of the electrical conductivity, due to the alignment of the MWCNTs in the flow direction. The study showed both higher conductivity (σ) values and lower p_c_ for the x- and y- than for the z-direction. Ramírez-Herrera et al. [21] made a similar study on compression molded PP/MWCNT plates, and the comparison of the electrical conductivity in two directions for plates with a thickness of 1.5 mm did not show large differences between the in-plane vs. through-plane values at concentrations of 5 wt% MWCNT (5.6E–05 S/cm vs. 1.5E–05 S/cm, respectively). Somehow, larger differences were observed at the higher concentration of 20 wt% MWCNT (6.1E–01 S/cm vs. 5.1E–02 S/cm, respectively). The study also shows in-plane conductivity values for “reprocessed” plates with a thickness of 0.1–0.2 mm, which is much higher than those of the original plates. Unfortunately, results in through-plane direction were not given. In both papers, two different devices for the measurements in the two directions have been used. However, for the comparability of the results, it is advantageous that the same equipment with a defined size of electrodes, current value and internal resistance can be used for the measurements in both directions. Since there are no commercially available devices, a device able to measure the electrical conductivity in two directions was developed and constructed (TWM1) at IPF Dresden (see details in the experimental part). The aim of the present study was to show the dependency of the electrical conductivity on the measuring direction for different polymer composite systems consisting of different polymer types, filler types, and concentrations, which were shaped by two different methods: compression molding and film extrusion. Such measurements were used to give at least qualitative information about the filler orientation in the respective sample direction. 

## 2. Materials and Methods

Commercially available polymer materials were used: poly (vinylidene fluoride) (PVDF) type Solef1006 (Solvay, Lyon, France) with a melt flow index (MFI) of 40 g/ 10 min at 2.16 kg loading (230 °C); polyamide 6 (PA6) Ultramid^®^ B27E (BASF, Mannheim, Germany); and acrylonitrile butadiene styrene (ABS) Cycolac^TM^ S570 (Sabic, Riyadh, Kingdom of Saudi Arabia) granules. As carbon fillers, 8 different types were selected. Among the group of carbon blacks (CB), a highly structured Ketjenblack EC600JD (Akzonobel, Amersfoort, The Netherlands) with a specific surface area (BET) of 1200 m^2^/g, a primary particle size d_50_ = 34 nm and an aggregate size of 30–100 nm was chosen. Three different types of carbon nanotubes were selected: singlewalled carbon nanotubes Tuball™ (OCSiAl S.a.r.l., Leudalange, Luxembourg) (SWCNT) having a mean diameter of 1.6 nm and length > 5 μm [28] (aspect ratio > 3125) and a BET of 500 m^2^/g; multiwalled CNTs CNT103 (Beijing Dk Nano technology, Beijing, China) (MWCNT) with diameters of ~8–15 nm, length ~50μm (aspect ratio: 6250–3333) and BET = 233 m^2^/g; and branched MWCNT CNS flakes with a 3 wt% poly (ethylene)glycol coating (Applied NanoStructured Solutions LLC, Baltimore, MD, USA) (b-MWCNT) with a diameter of 14 ± 4 nm and length of ~70μm (aspect ratio: ~5000) (according to the suppliers). In addition, graphite (G) and expanded graphite (EG) were applied. Among different graphite types, two SIGRACELL^®^ Graphite Additives with d_50_ particle sizes of 2 μm (here denoted as G-2 μm) and 4 μm (denoted as G-4 μm), and among expanded graphites, SIGRATHERM^®^ GFG5 (EG-5 μm) and GFG20 (EG-20 μm) with d_50_ particle sizes of 5 μm and 20 μm, respectively, were chosen, all supplied by SGL Carbon (Meitingen, Germany). The thicknesses of the G and EG platelets are not given by the supplier, so no absolute aspect ratio values can be given. However, assuming the same thickness in both EG samples, the aspect ratio in EG-20 μm is 4 times as large as in EG-5 μm. For G with two different sizes, it is expected that G-4 μm results in twice the aspect ratio as G-2 μm. The BET values of the G and EG fillers were determined with the Surface Area Analyser Autosorb iQ3 (Quantachrome Instruments, Boynton Beach, FL, USA). Values of 18, 20, 32, 27 m^2^/g for G-2 μm, G-4 μm, EG-5 μm and EG-20 μm, respectively, were measured.

Melt mixing was done on a small-scale by using a conical twin- screw compounder DSM15 microcompounder Xplore (Sittard, The Netherlands; volume: 15 cm^3^), with a circulating, closed system enabling adjustable mixing time. After drying for 1 h at 80 °C, the fillers and polymer granules (15 g input) were melt mixed for 10 min, at a temperature of 210 °C and a rotation speed of 200 rpm. The extruded strands were cut into granule-like sizes. In addition, few compositions were chosen for larger scale compounding, using a laboratory twin-screw extruder ZE 25 (KraussMaffei Berstorff GmbH, Munich, Germany) by applying a screw with a L/D ratio of 48. The mixing was done using polymer-filler premixtures at a temperature of 210 °C, a rotation speed of 200 rpm, and a throughput of 5–10 kg/h.

The granules obtained from the microcompounder and a control sample from the extruder were compression molded for electrical measurements to circular plates with a thickness of about 300 μm and a diameter of 6 cm, using the hot press PW40EH (Otto Paul Weber GmbH, Remshalden, Germany). The compression molding conditions were selected according to the polymer matrices and were: PVDF—temperature of 200 °C, compression time of 2.5 min, PA6—260 °C for 1.5 min, ABS—260 °C for 2.0 min at a pressure of 50 kN for all materials.

Cast film extrusion was performed with the PVDF granules obtained by twin-screw extrusion using a 30 mm single-screw extruder (DAVO GmbH, today Polyrema KG, Troisdorf, Germany), in combination with a cast film line (Dr. Collin GmbH, Maitenbeth, Germany). The width of a flat die was 30 cm, the gap width was set to 100 μm, and the melt temperatures were 230 °C, 260 °C, and 270 °C for composites with MWCNT, b-MWCNT and CB, respectively. The take-off velocity was set to ~3.2 m/min. Rolls of films with a width of ~22 cm, a thickness of 100 μm, and a length of approx. 22 m were achieved.

To evaluate the macrodispersion of the filler within the composites by transmission light microscopy (LM), thin sections with a thickness of 5 μm were cut from the microcompounder derived strands, with a filler content of 1.0 wt%, using the microtome RM2265 (Leica GmbH, Wetzlar, Germany) at room temperature. The sections were fixed on glass slides with the water-based embedding material, Aquatex (Merck, Darmstadt, Germany). The LM investigations were performed using a BX53M microscope combined with a DP71 camera (both Olympus Deutschland GmbH, Hamburg, Germany). 

The morphological characterization of the fillers and the composites was performed by scanning electron microscopy (SEM) on filler powders and extruded films using a Carl Zeiss Ultra plus microscope (Carl Zeiss Microscopy, Jena, Germany) with an SE2 detector or in charge contrast imaging (CCI) mode.

**Figure 1:**
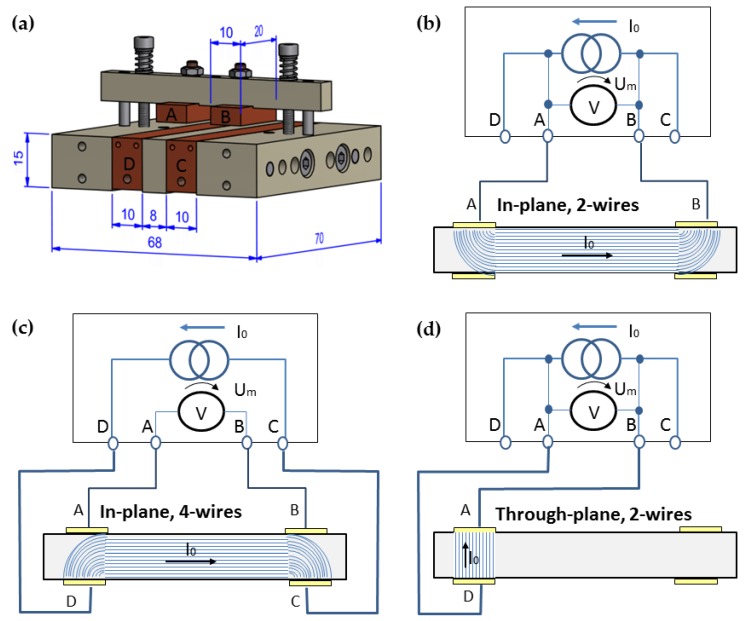
Device temperable resistance measurement (TWM1) for electrical volume resistance measurements in two directions, (**a**) schema, principle of (**b**) in-plane measurement with 2 wires, (**c**) or 4 wires, and (**d**) through-plane measurement.

For the electrical measurements, a device named TWM1 (in German: “Temperierbare Widerstandsmessung”- “temperable resistance measurement”), able to measure the electrical resistance of flat samples (x: 20–50 mm, y: 30–40 mm, z: up to ~5 mm) in two directions, was developed and constructed. It consists of four copper electrodes, two on the bottom side (70 mm × 10 mm × 15 mm) and two at the top (20 mm × 10 mm × 4 mm). The electrodes were mounted on poly (ether ether ketone) (PEEK) parts to ensure electrical isolation. The top-electrodes A and B shown in Figure 1a are held by a balk, which is pressed with the force of two springs on the lower electrodes C and D. The springs provide a nearly constant force on the sample, and the device is combined with the Keithley multimeter DMM2001 for measuring the resistance (Keithley Instruments, Solon, OH, USA). The schemas of the in-plane and through-plane measurements including the expected current flow lines are shown in Figure 1. The measurement sample area was constant and given by the size of the upper electrode (10 mm × 20 mm, see Figure 1a). The distance between the electrodes for the in-plane measurement was 8 mm (see Figure 1a). The size of the samples was 30 mm × 25 mm, and the sample thickness was ~300 μm for the plates and ~100 μm for the films. The filler orientation was analyzed using the ratio σ_in/th_ calculated as in-plane conductivity σ_in-plane_ (σ_in_) divided by through-plane conductivity σ_through-plane_ (σ_th_). The ratio σ_in/th_ is expected to increase with a more pronounced filler orientation in the processing direction. 

The in-plane measurements were accomplished, depending on the resistance values, with the 2-wire (>1 kΩ) or 4-wire (<1 kΩ) technique. All through-plane measurements were performed using the only available 2-wire technique. Given values are the mean of 4 measurements with standard deviations lower than 1% for the in-plane direction and about 5% for the through-plane direction. In order to reduce the interfacial resistance between the electrodes and the sample surface, conductive silver paste Electrodag 1415 (Agar Scientific, UK) was used. This paste is based on MEK/diacetone alcohol (2:1 by weight) solvent and does not influence the electrical conductivity of the sample itself. This paste was especially needed for the extruded films with high surface roughness (see Figure S1 and Table S1). Since the DMM2001 multimeter allows measurements of only moderate and low resistances, the electrometer 6517A, combined with the Measuring Device 8009 (both Keithley Instruments, Solon, OH, USA), was used in order to measure extremely high resistances (>1E+07 Ω, through-plane direction). Such measurements completed the percolation curves, including values at very low filler loadings. For this process, round samples with 60 mm diameters and thicknesses as given above were used. The values given are means of 4 measurements, and the standard deviation was about 2%. Those values were denoted with open symbols to distinguish them from those achieved by the TWM1.

The roughness of the plates and film surfaces was investigated using a confocal 3D microscope µsurf (Nanofocus, Oberhausen, Germany).

## 3. Results und Discussion

### 3.1. Morphology of Carbon Based Fillers

**Figure 2:**
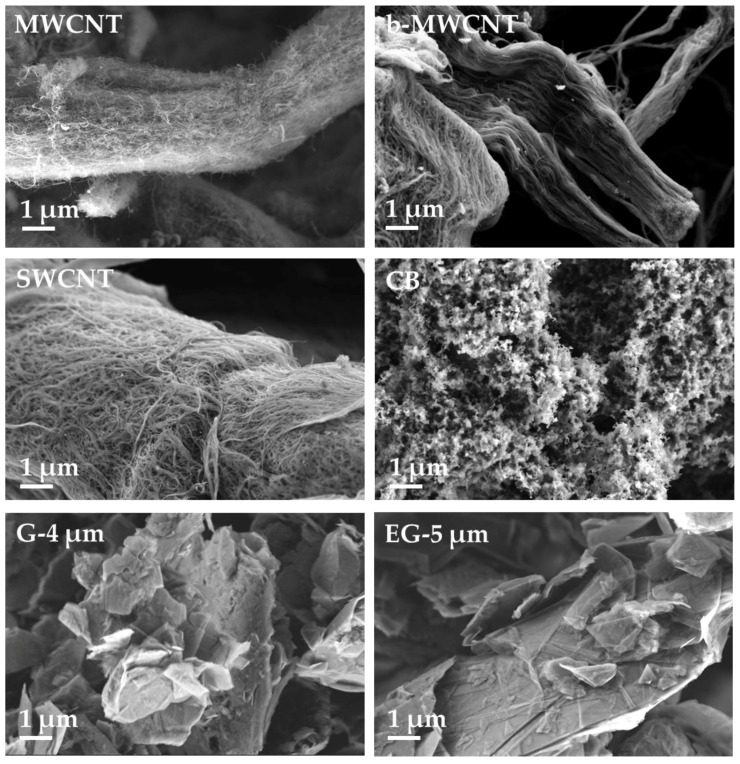
SEM images of structures of different kind of fillers. MWCNT, multiwalled carbon nanotubes; b-MWCNT, branched multiwalled carbon nanotubes; SWCNT, singlewalled carbon nanotubes; CB, carbon black; G, graphite; EG, expanded graphite.

The morphology of the carbon fillers was studied by SEM (Figure 2) at comparable magnification. The as-received powders of the MWCNT, b-MWCNT, and SWCNT illustrate yarn-like structures. Long nanotubes in the b-MWCNT and bundles of SWCNTs can be recognized. The highly structured form and primary particles of CB are very visible. Graphite (G-4 μm) and expanded graphite (EG-5 μm) do not show a significantly different appearance at the selected magnification. The slightly larger flake size of EG as compared to G can be seen.

### 3.2. Influence of the Carbon Filler Aspect Ratio on the Volume Conductivity of PVDF Composites

**Figure 3:**
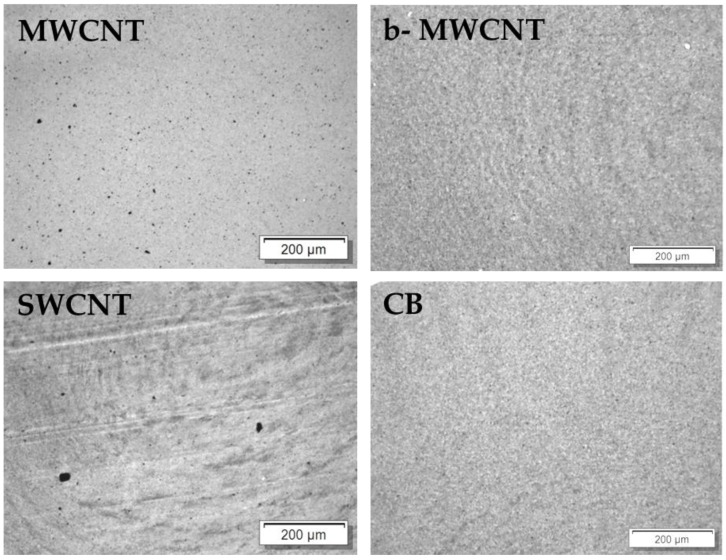
Transmission light microscopy (LM) images of poly (vinylidene fluoride) (PVDF) filled with 1.0 wt% of different kind of fillers.

**Figure 4:**
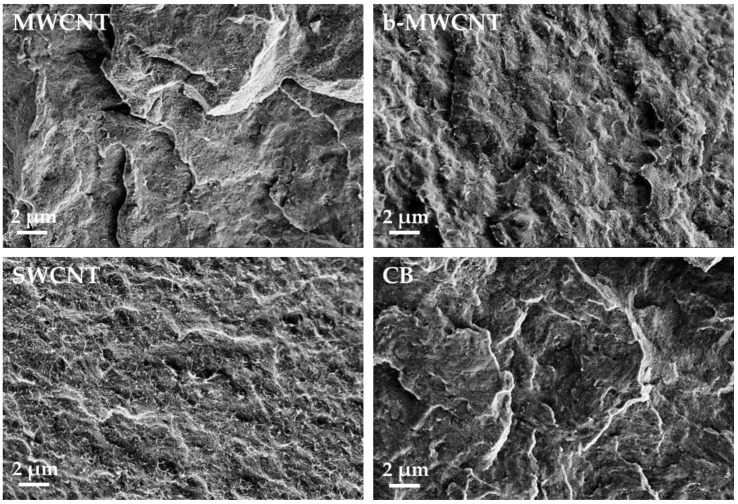
SEM micrographs of poly (vinylidene fluoride) (PVDF) filled with 1.0 wt% of different kind of fillers.

The influence of carbon fillers that have different aspect ratios on the electrical properties of PVDF composites was investigated on compression molded plates. The composites with CNTs and CB show a relatively good dispersion, as seen in the LM micrographs of samples with 1 wt% filler in Figure 3 and the SEM images of the CNT filled samples with 1 wt% in Figure 4. Only very small remaining agglomerates are seen for the MWCNT and SWCNT based samples (Figure 3). SEM micrographs shows the significantly higher length of the b-MWCNT in comparison to the SWCNT and MWCNT, which can be observed even in the composites after the melt-mixing (Figure 4).

**Figure 5:**
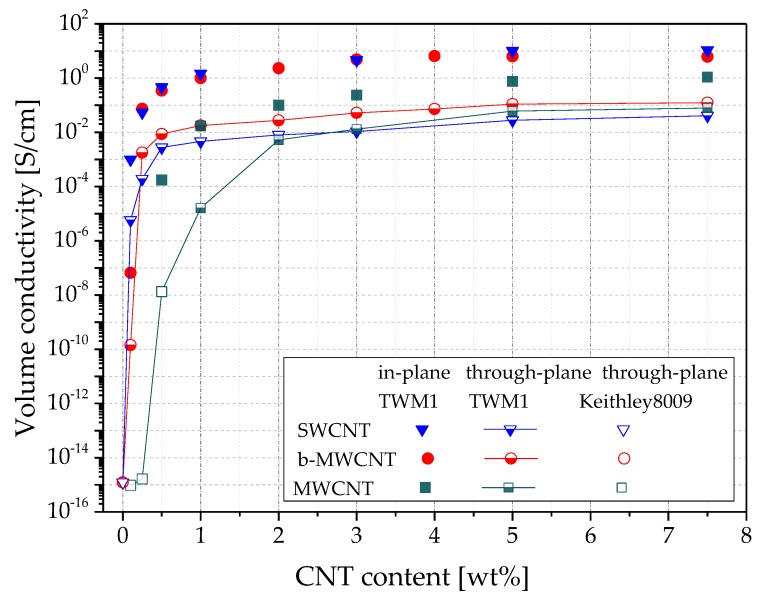
Volume conductivity vs. filler content of PVDF composites with CNTs with different morphology, measured in two directions on compression molded plates.

In Figure 5, the volume conductivities σ_in_ and σ_th_ at different filler contents of CNTs are shown, and in Table 1, the respective σ_in/th_– quotients, also including CB based samples, are shown. The in-plane values for b-MWCNT and SWCNT lay in the same range as the values found in [29].

The electrical percolation curves of the three CNT materials differ significantly, especially between SWCNT and b-MWCNT on the one hand, and MWCNT on the other hand (Figure 5). This difference is due to the fact, that all three CNT types differ from each other strongly in many parameters, such as diameter, length, purity grade, additives (in the case of b-MWCNT), chemical surface characteristics, and specific surface area. MWCNT possess two times lower specific surface area than SWCNTs (233 m^2^/g vs. 500 m^2^/g), but they have much larger mean tube diameters (15 nm vs. 1.6 nm). When compared to the other CNTs, b-MWCNT has a branched structure, which is assumed to promote the network structure formation in the composites and shows comparatively better dispersibility. As seen in Figure 3, b-MWCNT are well dispersed, whereas the composite with 1.0 wt% MWCNT shows some very small agglomerates and that with SWCNT some larger agglomerates.

For the CNTs, a very high difference between the electrical conductivity values measured in both directions was observed. At 1.0 wt% filler, the ratio σ_in/th_ is 1071 for MWCNT, 317 for SWCNT, and 56 for b-MWCNT. With increasing filler content, the ratio σ_in/th_ remains high for SWCNT (264 at 7.5 wt%) and decreases up to 50 for b-MWCNT and 14 for MWCNT at 7.5 wt%. The alignment of SWCNT in-plane seems to be strongest among all used CNTs, whereas the branched structure of the b-MWCNT promotes the development of a conducting network, resulting in higher conductivity values.

It can be assumed that the filler shape is the main parameter responsible for the filler orientation. Thus, the comparison of the quotients of the direction dependent conductivities seems to be appropriate. In contrast, the conductivity values of the composites with the three different CNT types only give limited information about the orientation degree. Lower σ_in/th_ quotients for b-MWCNT can be explained with the presence of branches in the CNT material, which helps to connect adjacent CNTs not only in-plane, but also occasionally through the thickness. The very high σ_in/th_ quotient for MWCNT, 1071 at 1.0 wt% loading, also indicates a very strong alignment for that type of CNTs. The sharp decrease to the much lower value σ_in/th_ = 19 already at 2.0 wt% shows the same concentration effect as with b-MWCNT. This is probably due to the higher aspect ratio of b-MWCNT and MWCNT compared to SWCNT.

As shown in Table 1, the quotients σ_in/th_ for the PVDF/CB composites are 3, 5, and 1 for 2.0 wt%, 3.0 wt%, and 7.5 wt% loading, respectively: significantly lower than those for PVDF/CNT composites. This finding indicates no orientation of the CB aggregates in the compression molded plates. The comparison at 1.0 wt% CB, and at loadings below that content, cannot be made, since the PVDF/1.0 wt% CB composite is not electrical conductive in any direction (σ below the percolation threshold).

**Figure 6:**
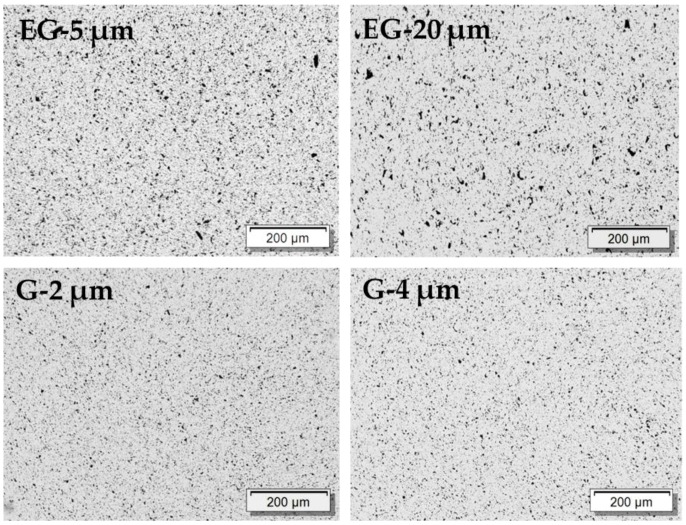
Transmission light microscopy images of PVDF/1.0 wt% composites filled with different kinds of expanded graphite (EG) and graphite (G).

**Figure 7:**
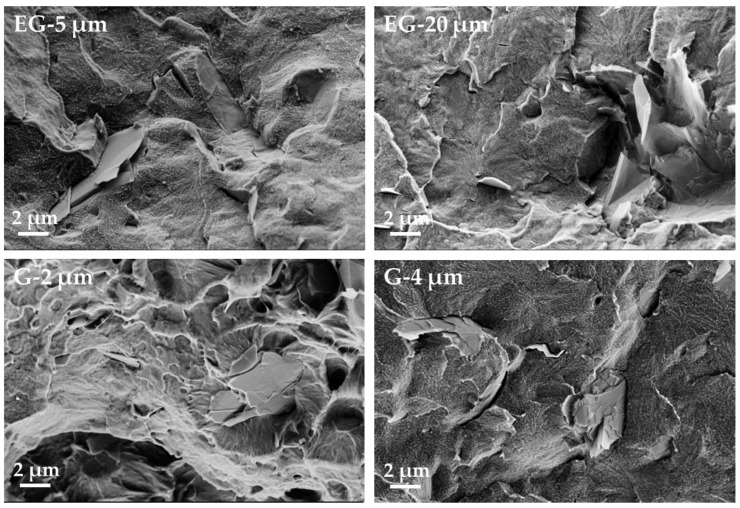
SEM micrographs of PVDF/1.0 wt% graphite composites filled with different kinds of expanded graphite (EG) and graphite (G).

**Figure 8:**
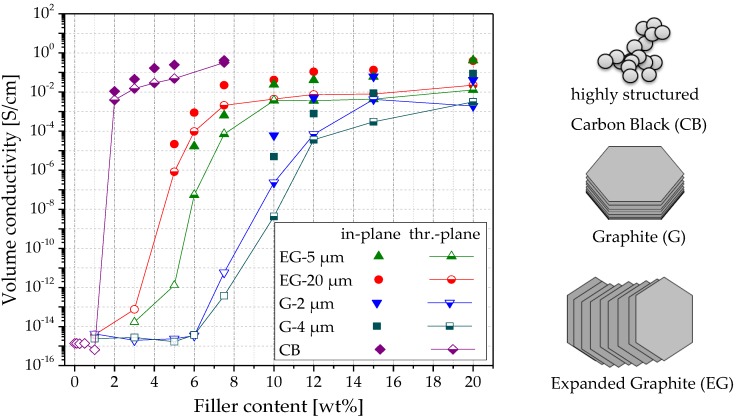
Volume conductivity vs. filler content for PVDF composites with carbon black (CB), expanded graphite (EG), and graphite (G), measured in two directions on compression molded plates. Open symbols measured using a Keithley 8009 measuring device.

The results using the composites with the filler graphite, G, and expanded graphite, EG, are shown in Figure 6, Figure 7 and Figure 8 and Table 2. The LM investigation of composites with 1 wt% filler (Figure 6) shows small graphite particles whose maximum sizes correspond to the initial sizes. In general, a good distribution can be seen. As compared to the CNTs (Figure 5), the electrical percolation thresholds of G and EG based composites are much higher, and the conductivities in both directions are much lower (Figure 8). This fact can be explained by the platelet-like shape of the EG and G fillers as compared to the rod-like shape of the CNTs. In addition, these materials have a much lower specific surface area, when compared to the highly structured CB (BET ~1200 m^2^/g, ~20 m^2^/g, ~30 m^2^/g for CB, G and EG, respectively). When comparing EG and G based composites, higher electrical conductivities and lower percolation thresholds are found for EG as filler. The EG with the larger particle size (EG-20 μm) leads to a percolation threshold (4–5 wt%) which is about 1 wt% lower than that of EG-5 μm (5–6 wt%). The two graphite types, which were smaller in mean diameter than the EG types, do not differ significantly in percolation thresholds and conductivity values; however, a slightly lower threshold is seen for G-2 μm. The comparison of EG-5 μm to G-4 μm, which both have very similar mean platelet sizes, shows a significantly lower percolation concentration of EG based composites (5–6 wt% vs. ~10 wt%). This difference is expected to be caused by the thinner plates in the expanded form of the graphite, resulting in a higher number of platelets at the same filling level and is also reflected in the higher measured BET values.

When comparing conductivities in two directions, the highest quotient σ_in/th_ was always found close to the respective percolation thresholds, which is comparable to the results for the PVDF/CNT composites. Among EG materials, a higher quotient was found for the smaller type (EG-5 μm, σ_in/th_= 309 at 6.0 wt% vs. EG-20 μm, σ_in/th_ = 26 at 5.0 wt%), and among G materials, the higher quotient was found for the larger type (G-4 μm, σ_in/th_ = 1165 at 10 wt% vs. G-2 μm, σ_in/th_ = 260 at 10 wt%). The reason for this result can be found in the fact that at 6 wt% loading, EG-5 μm is closer to its percolation threshold than EG-20 μm at 5 wt%. The same applies for the G samples at 10 wt% loading. G-4 μm is nearer to the percolation threshold of this sample than G-2 μm is to its percolation threshold.

At the higher filler contents (above 10 wt%), all σ_in/th_ quotients are similar and vary in a range between 9 and 33, indicating a comparatively low pronounced filler orientation. This result shows that the alignment behavior of plate-like graphite lies between those of CNTs, with rod-like morphology, and CB aggregates, with an aspect ratio close to one.

### 3.3. Influence of Polymer Matrix Type on Conductivity and Filler Orientation in SWCNT Based Composites

**Figure 9:**
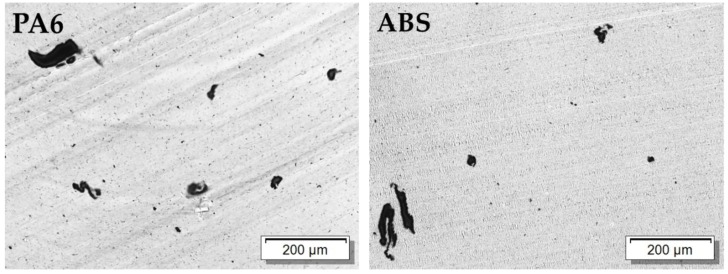
Transmission light microscopy images of polyamide 6 (PA6) and acrylonitrile butadiene styrene (ABS) filled with 1.0 wt% of SWCNT.

**Figure 10:**
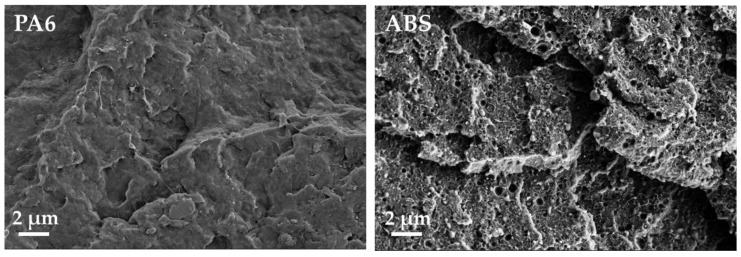
SEM micrographs of polyamide 6 (PA6) and acrylonitrile butadiene styrene (ABS) filled with 1.0 wt% of SWCNT.

**Figure 11:**
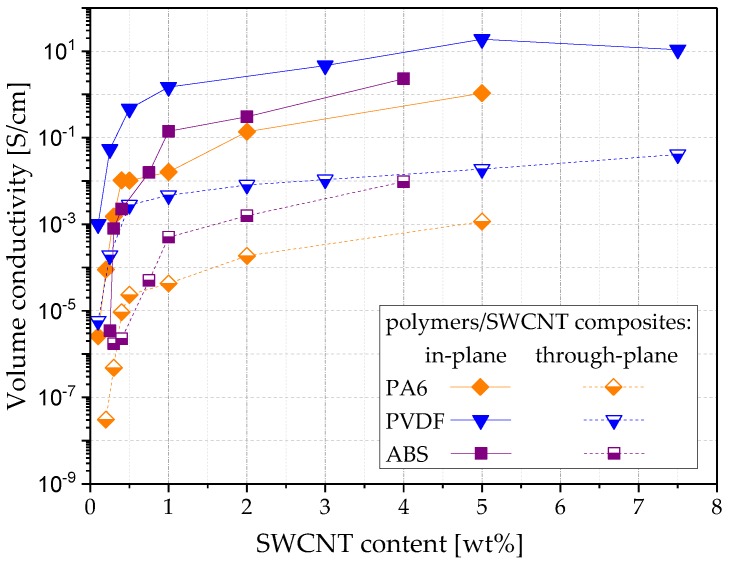
Volume conductivity vs. filler content for SWCNT composites based on PA6, PVDF or ABS matrix, measured in two directions on compression molded plates.

The results obtained on SWCNT composites based on different polymer matrix types are shown in Figure 9, Figure 10 and Figure 11 and Table 3. The light microscopy images (Figure 3 and Figure 9) illustrate the best SWCNT dispersion for PVDF as a matrix, followed by PA6 and ABS. In all composites, remaining agglomerates can be seen. The electrical percolation thresholds p_c_ of the used SWCNT were found to be below 0.1 wt% for PVDF [29] and PA6, and below 0.25 wt% for ABS based composites (Figure 11). The conductivity values are the highest for PVDF, followed by ABS and PA6 based composites. In a wide range of filler content (≥1.0 wt%, clearly above electrical percolation threshold), the values of σ_in/th_ vary between 200 and 500 (Table 3). It can be concluded that the filler orientation is nearly independent of the kind of polymer matrix and is also present at higher filler loadings. Additionally, for PA6/SWCNT composites, filler contents near the electrical percolation threshold were studied. At 0.2–0.3 wt% SWCNT content, a ratio σ_in/th_ of ~3000 was measured, indicating a very strong in-plane filler orientation. The ratio σ_in/th_ decreased to 1120 and 440 at 0.4 wt% and 0.5 wt% loading, respectively.

Moreover, for ABS/SWCNT composites the σ_in/th_ values near the percolation threshold of 0.25 wt% were high (455 @ 0.3 wt%, 997 @ 0.4 wt%), indicating a high filler orientation. However, the orientation effect was lower compared to PA6/SWCNT composites.

### 3.4. Influence of Shaping Procedure on Volume Conductivity and Filler Orientation inPVDF Based Composites

The influence of the film extrusion process on electrical conductivity and filler orientation was investigated in comparison to the values obtained on the compression molded plates (Table 4 and Table 5). The same PVDF based composite material filled with b-MWCNT, MWCNT, and CB was used for both shaping procedures. Whereas the pressed plates had thicknesses of about 300 μm, extruded films were ~100 μm thick. While, for the plates, the conductivity was measured in two directions (assuming random orientation in all in-plane direction), for the films, three directions were studied. Based on the results shown before, for this study, only lower filler contents were selected, at which higher differences in conductivities between pressed plates and extruded films could be observed. This result is due to the fact that, at concentrations slightly above the percolation threshold, the orientation phenomenon is not shielded yet by the filler concentration effect. 

**Figure 12:**
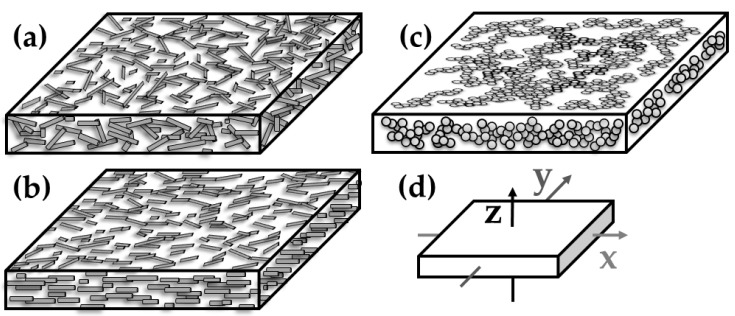
Schemes of filler orientation: (**a**) CNTs in compression molded plates, (**b**) CNTs in extruded films in extrusion direction x, (**c**) CB in plates and films, (**d**) schema of measurement directions for films: z= through-plane; x = in-plane, **║** to the extrusion direction; y = in-plane, **⊥** to extrusion direction.

At the content of 1.0 wt% b-MWCNT, the in-plane conductivity for pressed plates is slightly higher than for films by the factors 6, 2, and 7 in z, x and y direction, respectively (Table 4). The differences are lower for 2.0 wt% b-MWCNT (factors: 2, 1, and 3 in the z, x, and y directions, respectively). The direct comparison of the measurement directions using the quotients σ_in/th_ for plates and films (Table 5) clearly shows the effect of the shaping type on the b-MWCNT orientation. The quotient σ_in x/th_ is 166 for films and 56 for plates. Whereas the in-plane alignment of the b-MWCNT in the plates is of moderate nature, the melt flow and take-off forces during the film extrusion cause a strong in-plane alignment. This is schematically illustrated in Figure 12, where a cross-section of plates and films with CNTs and CB are shown next to the direction assignment. For films, two in-plane conductivities have to be distinguished accordingly; these conductivities are to be measured in the extrusion direction (x) and perpendicular to the extrusion (y). This quotient, σ_in x/in y_, is 4 for the films of PVDF, with 1 or 2.0 wt% b-MWCNT, which shows a clear filler orientation in the extrusion direction. This is induced by both melt flow through the die and take-off forces stretching the film during solidification. 

**Figure 13:**
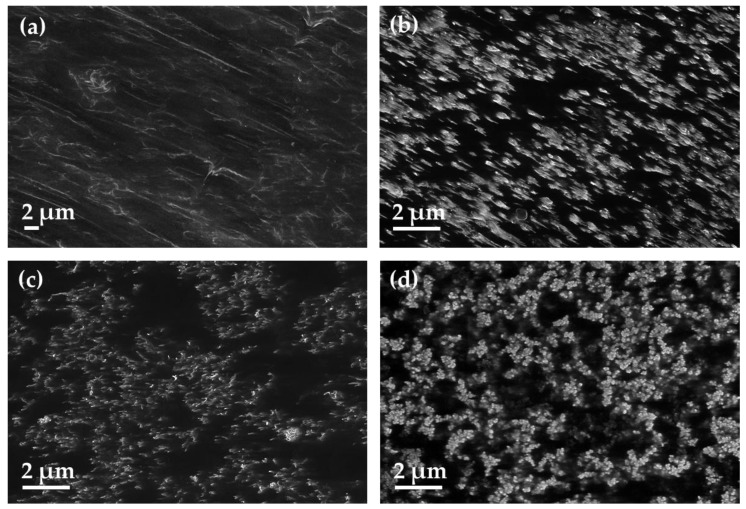
SEM-charge contrast imaging (CCI) mode micrographs of extruded films of PVDF composites: (**a**) 2.0 wt% b-MWCNT, surface and (**b**) 2.0 wt% b-MWCNT, cross-section; (**c**) 2.0 wt% MWCNT, cross-section; (**d**) 3.0 wt% CB, cross-section.

The PVDF composites filled with b-MWCNT were compared to those with the other fillers, MWCNT and CB, regarding their composite morphology (Figure 3 and Figure 4) and electrical properties (Table 4 and Table 5). The SEM images taken in charge contrast imaging (CCI) mode (Figure 13) illustrate the strong alignment of the b-MWCNT and MWCNT in the extrusion direction (a–c), whereas no alignment is observed in the case of carbon black (d). Interestingly, the micrographs taken at the surface of the film show strong alignment, whereas the cross-sections of composites with b-MWCNT and MWCNT show a more agglomerated filler structure, in which the CNTs form regular clusters that can also promote higher electrical conductivity.

At 2.0 wt% loading, a direct comparison between composites with b-MWCNT and MWCNT was performed. Since the conductivity in both directions of the PVDF/2.0 wt% composites is already much lower in the pressed plates when using MWCNT compared to b-MWCNT (z: 5.25E-03 S/cm vs. 2.7E−02 S/cm and x = y: 1.0 E−01 vs. 2.3E+00 for MWCNT and b-MWCNT, respectively), a much lower bridging potential of the nanotubes and a weaker nanotube network can be assumed for the MWCNT. This phenomenon can explain the higher conductivity quotients shown in Table 4, which illustrates a stronger alignment of the non-branched MWCNT in the films. Almost no alignment effect caused by film extrusion can be observed for the PVDF/4.0 wt% CB composite, while very similar conductivities in each direction are measured for the plate and the film. The quotient σ_in x/in y_ is close to 1 and σ_in x/th_ and σ_in y/th_ are 5 and 4, respectively, for the film. This is very close to the σ_in x=y/th_ of 6 for the plate (see Table 5) and shows that the orientation of CB aggregates in the plates and in the film is comparable (Figure 12c). This means that the shaping procedure has no effect on the conductivity of the PVDF/CB composites. 

## 4. Conclusions

Using newly developed equipment, measurements of electrical conductivity in up to 3 dimensions were performed on compression molded plates and extruded thin films. Melt mixed composites with varying filler content in the range between 0.1 and 7.5 wt% were compression molded to plates for all filler types, and films based on PVDF composites were extruded at filler contents between 1.0 and 2.0 for CNTs and 4.0 wt% for CB. While the plates were measured in two directions (x and z), the films were measured in three directions (in-plane in extrusion direction x, in-plane perpendicular to extrusion y and through-plane z). The quotients σ_in/th_ and σ_in x/y_ were used to gain information about the filler alignment in the different samples. Thereby, different filler types including CNTs, graphite, expanded graphite and carbon black, and three matrices, namely, PVDF, PA6, and ABS, were used. 

The values of electrical conductivity and the quotients indicate a strong alignment of all anisotropic fillers in the in-plane direction in compression molded plates. The higher the σ_in/th_ quotient, the higher the in-plane alignment of these fillers. The effect of alignment is most significant when the filler content is slightly above the percolation threshold, and alignment is most strongly noted for the non-branched MWCNT and SWCNT. Branched MWCNT show moderate alignment and higher conductivities in the through-plane direction than the other CNT types at all concentrations, which can be explained by the branches, promoting the development of the conducting network. Carbon black causes a much higher electrical percolation threshold than CNTs due to its low aspect ratio close to 1. On the other hand, this shape is a reason for a very low alignment of its aggregates in compression molded plates, indicated by a very low σ_in/th_ quotients. The much higher electrical percolation threshold and the lower electrical conductivities for G and EG compared to CNTs and CB in both directions can be explained by a much higher size of the G and EG particles and much lower specific surface area. When comparing electrical conductivities in two directions for the investigated EG and G filler, large differences between them are measured near the percolation threshold, but at higher contents, all quotients are similar and vary in the range σ_in/th_ ~10–30. This result shows that the alignment behavior of graphite (plate form) lies between that of CNTs with rod-like shape and high aspect ratio and CB aggregates with an aspect ratio close to one. The data showed no influence of the matrix type on the changes in filler orientation.

The investigations on the influence of film extrusion showed large differences between the compression molded plates and films at lower filler concentrations. At a content of 1.0 wt% b-MWCNT the quotient σ_in x/th_ was 166 for films and 56 for plates. This result shows that although the planar orientation of the b-MWCNT in the plates is moderate, the melt flow and take-off forces during film extrusion produce a strong orientation. An additional alignment within the in-plane direction is observed in the films and is expressed by the quotient of the two in-plane conductivities σ_in x/in y_. This quotient is 4 for PVDF/2.0 wt% b-MWCNT, indicating a clear CNT alignment in the melt flow or x-direction. There are almost no alignment effects observed for the PVDF/4.0 wt% CB due to film extrusion, as very similar conductivities are measured for the film in each direction, which also corresponds to the values of the compression molded plates. This means that the shaping method has no influence on the conductivity of the composites if fillers with an aspect ratio close to 1, such as CB, are used.

The method used in this study to characterize filler orientation by the ratio of electrical conductivities measured in different directions is easily performed. However, this method is not able to give the absolute orientation factors of the fillers. Such information could be added, e.g., when using quantification of TEM micrographs [30,31,32,33]. The degree of filler orientation of the CNTs could be also determined by polarized RAMAN spectroscopy or wide-angle X-ray diffraction (WAXD) [34,35].

## Figures and Tables

**Table 1:** Quotient of in-/through-plane σ values (σ_in/th_) of PVDF/carbon fillers composites, compression molded plates.

Filler Content	σ_in/th_ [-]			
	SWCNT	MWCNT	b-MWCNTs	CB
0.1	173	n.c.	466	n.c.
0.25	284	n.c.	41	n.c.
0.5	167	o.m.r.	40	n.c.
1.0	317	1071	56	n.c.
2.0	-	19	84	3
3.0	430	18	94	5
5.0	363	13	58	5
7.0	263	14	50	1

n.c., not electrically conductive. o.m.r., out of the measuring range of the equipment in through-plane direction.

**Table 2:** Quotient of in-/through-plane σ values (σ_in/th_) of PVDF/G and EG composites, measured in two directions on compression molded plates.

Filler Content	σ_in/th_ [-]			
	EG-5 μm	EG-20 μm	G-2 μm	G-4 μm
**5.0**	n.c.	26	n.c.	n.c.
**6.0**	309	9	n.c.	n.c.
**7.5**	9	11	n.c.	n.c.
**10.0**	7	9	260	1165
**12.0**	11	15	74	22
**15.0**	14	17	15	29
**20.0**	33	18	21	28

n.c., not electrically conductive.

**Table 3:** Quotient of in-/through-plane σ values (σ_in/th_) of different matrix composites with SWCNTs, measured in two directions on compression molded plates.

SWCNT [wt%]	σ_in/th_ in PVDF	σ_in/th_ in PA6	σ_in/th_ in ABS
0.1	173	o.m.r.	-
0.2	-	2927	-
0.25	284	-	o.m.r.
0.3	-	3147	455
0.4	-	1116	997
0.5	167	436	-
0.75	-	-	314
1.0	317	376	281
2.0	-	741	194
3.0	430	-	-
4.0		-	239
5.0	363	931	-
7.5	263	-	--

o.m.r., out of the measuring range of the equipment in through-plane direction.

**Table 4:** Comparison of the electrical conductivity of PVDF/carbon filler composites shaped into plates and extruded films and conductivity quotients σ_plate/film_ in x = y (for plates) or x, y (for films) and z (through-plane) directions.

Filler Content	σ_th_^*^ [S/cm]		σ_in (x*= y*)_ [S/cm]	σ_in__x_^*^ [S/cm]	σ_in__y_^*^ [S/cm]	σ_th plate/film_	σ_in plate/x film_	σ_in plate/y film_
	Plates	Films	Plates	Films				
1.0 wt% b-MWCNT	1.8E-02	3.2E-03	1.0E+00	5.4E-01	1.4E-01	6	2	7
2.0 wt% b-MWCNT	2.7E-02	1.7E-02	2.3E+00	2.5E+00	6.9E-01	2	1	3
2.0 wt% MWCNT	5.25E-3	8.2E-04	1.0E-01	5.1E-03	2.9E-03	6	9	16
4.0 wt% CB	2.9E-02	6.1E-02	1.7E-01	2.8E-01	2.4E-01	0.5	1	1

* z= through-plane; x = in-plane, ║to extrusion direction; y = in-plane, ⊥ to extrusion direction.

**Table 5:** Comparison of the σ quotients of electrical conductivities measured in two and three directions for plates and films.

Filler Content	Plates		Films		
	σ_in x=y/z_	σ_in x/in y_	σ_in x/th_	σ_in y/th_	σ_in x/in y_
1.0 wt% b-MWCNT	56	1	166	42	4
2.0 wt% b-MWCNT	84	1	144	40	4
2.0 wt% MWCNT	18	1	6	3	2
4.0 wt% CB	6	1	5	4	1

## Supplementary Materials

The following are available online at https://www.mdpi.com/2073-4360/11/4/591/s1, Figure S1: Roughness profiles achieved using 3D-Confocal microscopy µsurf expert (a) Al film; (b) PVDF/2.0 wt% b-MWCNT compression molded plate; (c) PVDF/2.0 wt% b-MWCNT extruded film; (d) PVDF/4.0 wt% CB extruded film. e, Table S1: Selected parameters for the characterization of surface topography by confocal microscopy.
