# Supplementary material for: Direction Dependent Electrical Conductivity of Polymer/Carbon Filler Composites
Source: Polymers (Basel). 2019 Apr 1;11(4):591. doi: 10.3390/polym11040591 (PMC6523830; doi:10.3390/polym11040591)
Supplement: Supplementary file 1 [file polymers-11-00591-s001.pdf]

polymers

Supplementary Materials

Roughness Measurements

When measuring the conductivity of samples with very low resistance, it is necessary to paint the contact points with conductive silver paste in order to reduce the contact resistance between the sample and the electrode. With such samples, the contact resistance can be higher than the actual resistance, which can significantly change the measurement result. The rougher the sample surface, the higher the contact resistance. Therefore, the roughness of selected samples was investigated and compared.

Figure S1 and Table S1 show roughness profiles and parameters measured with a confocal microscope on aluminum (Al) film and PVDF composite plates and films with two different fillers: CB and b-MWCNT. The results show a smooth surface for the Al film and the PVDF/2.0wt % b-MWCNT plate, while the highest roughness was found for the PVDF/2.0 wt % b-MWCNT film. This indicates that especially with extruded films an improvement of the contacts by silver paint is needed in order to obtain reliable conductivity results.

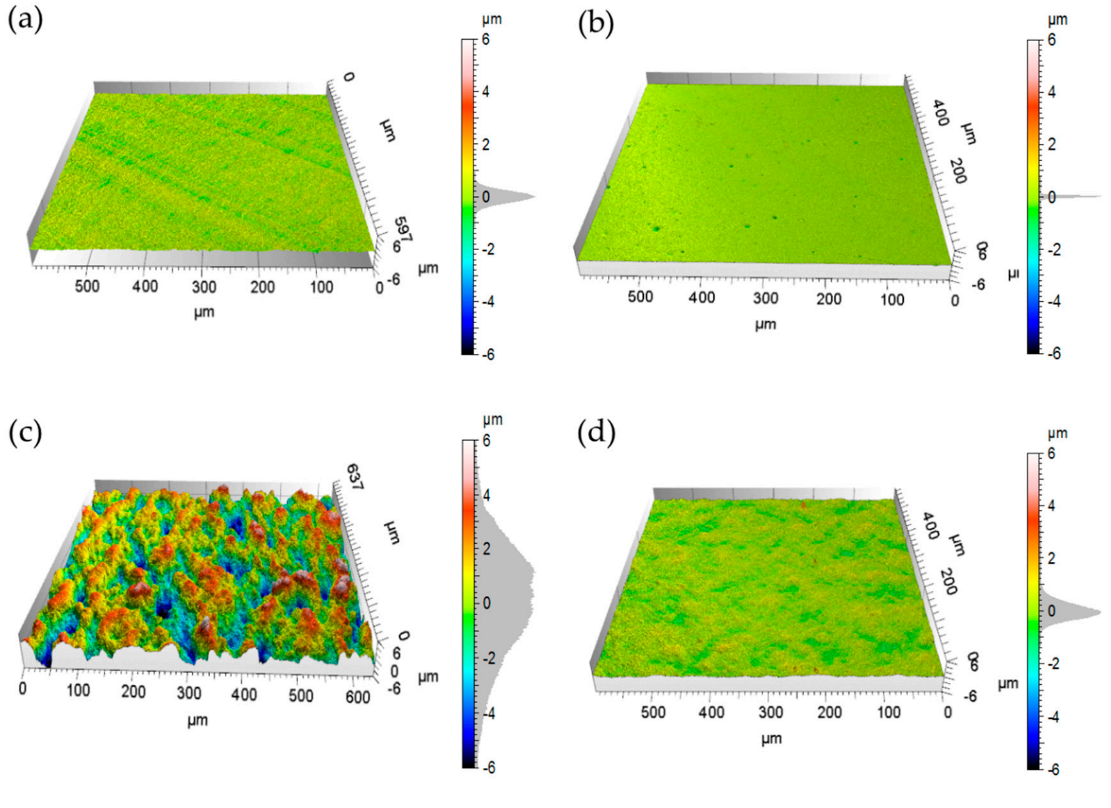

Figure 1. Roughness profiles achieved using 3D-Confocal microscopy µsurf expert a) Al film; b) PVDF/2.0 wt % b-MWCNT compression molded plate; c) PVDF/2.0 wt % b-MWCNT extruded film; d) PVDF/4.0 wt % CB extruded film.

Table 1. Selected parameters for the characterization of surface topography by confocal microscopy.

**Area roughness parameter**	**Adequate 2D- Scheme with 2D Parameters**	**Al (a)**	**PVDF Based Composites** 2.0wt% b-MWCNT (b)	**PVDF Based Composites** 2.0wt% b-MWCNT (c)	**PVDF Based Composites** 4.0wt% CB (d)
		**film**	**plate**	**film**	
S_a [μm] - the difference in height of each point compared to the arithmetical mean of the surface	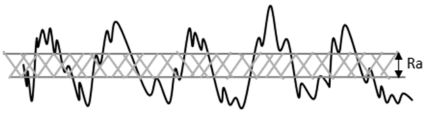	0.178	0.038	1.20	0.244
S_p [μm] - the height of the highest peak within the defined area	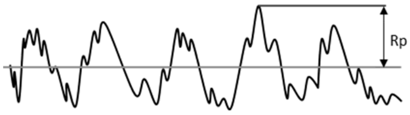	0.958	0.753	6.18	3.39
S_{dr} [%] - the percentage of the surface area contributed by the texture as compared to the planar area.	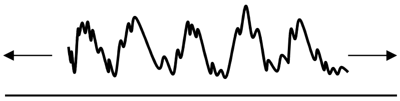	2.30	0.17	4.45	1.57
